# Complementary and Alternative Medicine for Premature Ovarian Insufficiency: A Review of Utilization and Mechanisms

**DOI:** 10.1155/2022/9053930

**Published:** 2022-04-01

**Authors:** Yang Fu, Dan-Ni Ding, Ying Shen, Li-Yan Jia, Meng-Yu Yan, Wei Wei, Chang-Hong Song, Feng-Juan Han

**Affiliations:** ^1^Department of Gynecology, The First Affiliated Hospital of Heilongjiang University of Chinese Medicine, Harbin 150040, China; ^2^Heilongjiang University of Chinese Medicine, First Clinical Medical College, Harbin 150040, China

## Abstract

Premature ovarian insufficiency (POI) is defined as a decline in ovarian function before the age of 40 and is one of the leading causes of infertility in women. The etiology is complex, and the pathogenesis is not clear. The main treatment is hormone replacement therapy, but a growing body of data confirms that such treatment can increase the risk of endometrial disease and cardiovascular disease. Complementary and alternative medicine (CAM) has been widely used in patients with POI due to its limited adverse reactions and high efficiency. According to literature reports, CAM therapy for POI mainly includes traditional Chinese medicine, acupuncture, psychotherapy, dietary supplements, and exercise therapy. This article reviews the application of CAM in the treatment of POI and attempts to determine the therapeutic effects and the mechanisms behind these effects based on existing clinical and experimental studies in order to provide theoretical support for the treatment of POI.

## 1. Introduction

**Figure 1:**
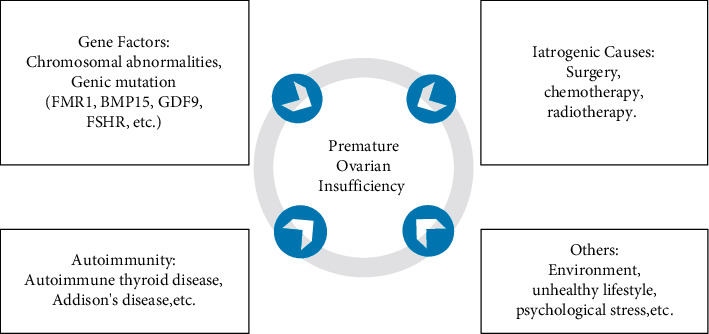
Etiologies of POI.

Premature ovarian insufficiency (POI) refers to a decline of ovarian function before the age of 40 and is one of the major causes of female infertility [1]. It is biochemically characterized by elevated gonadotropin levels and low estradiol (E_2_) levels, which lead to menstrual disturbance, induce menopausal symptoms such as hot flashes, night sweats, and insomnia, and increase the risk of decreased bone mineral density and cardiovascular disease [2]. The incidence of POI is approximately 1%–5% [3, 4], and its diagnosis should be based on the presence of menstrual disturbances with biochemical confirmation. According to the guidelines for POI developed by the European Society of Human Reproduction and Embryology (ESHRE), the diagnostic criteria are as follows: age <40 years, oligo/amenorrhea for at least 4 months, and elevated follicle-stimulating hormone (FSH) levels >25 mIU/ml on two occasions >4 weeks apart [5]. The exact etiologies of POI are still unclear, but they appear to include gene factors, iatrogenic causes, autoimmunity, unhealthy lifestyle, and psychological stress [6, 7] (Figure 1). Currently, the main way to treat POI is hormone replacement therapy (HRT), which can ameliorate the clinical complications caused by low estrogen but has no obvious effect on improving ovarian function or fertility [2, 4]. In addition, long-term HRT may increase the risk of endometrial disease, breast disease, and thrombotic disorders [8–11]. Therefore, there is an urgent need to develop better strategies for treating POI.

Complementary and alternative medicine (CAM) refers to various health care and treatment systems that are independent of western medicine. It has the advantages of being natural, convenient, and affordable and is widely used in many countries [12]. A large number of studies have shown that the use of CAM, including Chinese herbal medicine, acupuncture, moxibustion, psychotherapy, dietary supplements, and exercise therapy, can effectively treat POI (Table 1) with fewer adverse reactions. This review summarizes the research progress of CAM in the treatment of POI and analyzes the potential mechanism in order to provide more accurate evidence for its clinical application.

## 2. Search Strategy and Selection Criteria

A review of the literature was conducted to investigate the use of CAM to treat POI. We searched the PubMed, Web of Science, Embase, CNKI, and VIP databases using the keywords “Premature ovarian insufficiency,” “Traditional Chinese Medicine,” “Complementary and alternative medicine,” “Herbal Extracts,” and “Acupuncture and moxibustion”. References from relevant articles published between 1990 and 2021 were analyzed, and references in the identified studies were also searched. The search yielded 2,591 articles, of which 143 articles were deemed potentially relevant. Among them, there were 56 articles on Chinese herbal products, 42 articles on acupuncture and moxibustion, 7 articles on psychotherapy, 17 articles on dietary supplements, and 9 articles on exercise therapy. Articles were excluded due to various reasons, including selection bias, detection bias, reporting bias, and other possible sources of bias.

## 3. Herbal Products

Herbal products refer to compound preparations composed of two or more herbs that are processed into boiled decoctions, herbal extracts, medicinal gels/jellies, pills, or capsules (patent medicines). Tables 2 and 3 list the most commonly used herbs for treating POI. Their potential mechanisms include (1) inhibiting the secretion of the gonadotropin-releasing hormone through negative feedback regulation in order to maintain the normal menstrual cycle, (2) increasing the number of primordial follicles, reducing the number of atretic follicles, and promoting the growth of granulosa cells, (3) inhibiting or promoting the expression of related pathways and proteins and changing the apoptotic state of ovarian cells, and (4) reducing the level of proinflammatory cytokines and enhancing immune responses.

### 3.1. Herbal Decoction Therapies

There are several common decoctions used to treat POI in China, including Bushen Culuan Decoction (BCD), Bushen Huoxue Decoction (BHD), Huyang Yangkun Recipe (HYR), Erxian Decoction (EXD), and Huluan Decoction (HLD). Many clinical and experimental studies have confirmed that Chinese herbal decoctions play an important role in the treatment of POI.

#### 3.1.1. BCD

BCD is an empirical decoction that can restore normal serum sex hormone levels in mouse models of POI, increase the antral follicle count (AFC) and developed follicle count, and decrease the atretic follicle count. It can also downregulate caspase-3 protein expression and upregulate bone morphogenetic protein-7 (BMP-7) protein expression, thereby decreasing the granulosa cell apoptosis rate and maintaining ovarian function. The possible regulatory signaling pathways include the Smad pathway and NF-*κ*B pathway [22]. Two randomized controlled trials (RCTs) showed that after three months of treatment the total effective rate and pregnancy rate of BCD were better than the combination of E_2_ valerate + clomiphene + progesterone or the combination of climen + clomiphene [13, 23]. BCD has proven to be safe and effective for clinical use in treating infertility due to POI.

#### 3.1.2. BHD

BHD has been widely used to treat various disorders in China since the Qing dynasty. In traditional Chinese medicine (TCM), most doctors agree that the main pathogenesis of POI is kidney deficiency and blood stasis, so BHD is also extensively applied in treating POI [24]. More and more studies have shown that chronic stress is an important cause of decreased ovarian function and reproductive endocrine disorders [25–27], and BHD can restore serum levels of follicle-stimulating hormone (FSH) and anti-Müllerian hormone (AMH) in POI rats induced by the stress hormone corticosterone and can reduce follicular atresia and improve ovarian function. A mechanistic study also showed that BHD might regulate the expression of Np4 and Angptl 4 to improve corticosterone-induced POI [28]. In addition, BHD can also reduce the infiltration of inflammatory cells and the formation of zona pellucida in the ovaries of POI mice, reverse the abnormal sex hormone state, downregulate serum AzpAb levels, activate the Keap1/Nrf2/ARE signaling pathway, and increase the activity of the downstream antioxidant enzymes superoxide dismutase (SOD), heme oxygenase-1 (HO-1), and NAD(P)H: quinone oxidoreductase 1. Therefore, BHD may improve ovarian function in POI mice, reduce oxidative stress, and regulate the immune system through the Keap1/Nrf2/ARE signaling pathway [29,30]. In a clinical study, Zhong et al. [14] selected 40 patients who were randomly divided equally into the treatment group (BHD) and control group (HRT with E_2_ valerate and progesterone) for 3 months. The total effective rate of the treatment group was 100.0%, which was significantly higher than 70.0% in the control group.

#### 3.1.3. HYR

HYR was developed based on the theory of TCM as well as clinical experience. According to TCM theory, HYR is mainly used to treat POI due to spleen and kidney deficiency. HYR may slow down the atresia of follicles and enable more follicles to mature by decreasing the overexpression of aquaporins caused by 4-vinylcyclohexene diepoxide in order to regulate the expression of the apoptosis signaling pathway, especially the Bcl-2 family of proteins, in the ovary [31, 32]. Yang [15] treated 110 patients with POI with HYR or dehydroepiandrosterone, and the total effective rates were 94.55% and 85.45%, respectively. At the same time, the menstrual recovery rate of patients with mild and severe menses in the experimental group (HYR) was higher than that in the control group (dehydroepiandrosterone) (*P* < 0.05). Furthermore, transplantation of embryonic stem cells (ESCs) has great potential for improving POI, and studies have confirmed that HYR promotes the treatment effect of ESCs. The combination of HYR and ESCs might promote follicle development by inhibiting the activity of the TGF-*β*1/TAK1 pathway [33].

#### 3.1.4. EXD

EXD contains curculigo, radix morindae, angelica, and cornu cervi and is a common decoction for treating POI. The beneficial effects of EXD in POI are probably exerted via regulation of the immune system, modulation of estrogen levels, and antioxidative activities, and EXD may act in a synergistic or cooperative manner with other therapeutic agents [34]. Wu and Liu [35] found that EXD enhanced the expression of forkhead protein 3 (FoxP3) in POI model mice and activated CD4^+^CD25^+^ regulatory T cells (Tregs), thereby regulating immune function and preventing the occurrence of POI. After 3 months of treatment with EXD, the total effective rate in the 40 POI patients was 95% compared to 77.5% in the HRT control group, and the EXD group had significantly reduced TCM syndrome scores and significantly increased quality of life scores [16]. Yuan et al. [36] treated POI patients with EXD and reported an overall efficiency of 93.33%.

#### 3.1.5. HLD

HLD is an empirical decoction, including *Eucommia ulmoides*, *Cuscuta*, and other kidney-tonifying Chinese medicines. Eucommia ulmoides can inhibit the expression of T lymphocytes and reduce the level of proinflammatory cytokines so as to regulate immune homeostasis, improve immunity, and treat POI [37]. Shang [38] used HLD to treat immune-induced POI mice and found that it could regulate immunity by restoring the balance of Th17 and Treg cells, thereby promoting follicle formation and improving ovarian function. Deng [39] found that HLD could boost the activity of the SIRT1/NF-*κ*B/p53/p21 pathway in ovarian cells, thus changing the apoptotic state, increasing the numbers of growing and mature follicles, and reducing the number of atretic follicles. It could also significantly reverse the aging process and improve ovarian function. A random single-blind clinical observation of 60 POI patients showed that the symptoms of 83.3% and 66.7% patients were alleviated in the HLD treatment and the Femoston treatment groups, respectively [17]. HLD thus appears to be effective in treating POI and should be considered for clinical applications.

#### 3.1.6. Other Herbal Decoction Therapies

In China, several herbal mixtures are used to treat POI and have demonstrated positive effects. For example, Yishen Yangluan Decoction can regulate the PI3K/AKT signaling pathway, upregulate the expression of Bcl-2, and inhibit the expression of Bax protein in order to reduce oocyte apoptosis, alleviate the clinical symptoms of POI patients, and improve the ovarian reserve [40]. The results of a Danggui Buxue Decoction intervention in a rat model of POI showed that it might inhibit Foxo3a by upregulating Jak2, thereby mediating Bcl-2 family activities and inhibiting apoptosis in ovarian cells [41]. Moreover, after 6 months of oral treatment with Wumei Pill in 73 POI patients, their ovulation rate and positive pregnancy rate were significantly higher than those in the HRT group (47.3% vs. 39.1% and 29.5% vs. 20.1%, respectively) [42]. Yukun Decoction combined with E_2_ valerate tablets in the treatment of kidney deficiency and liver depression associated with POI can reduce FSH and LH levels and increase the level of E_2_, the AFC, the thickness of the endometrium, the diameter of the ovary, and the peak systolic velocity [43, 44]. Yijing Decoction has also been shown to be better than western medicine at reducing POI symptoms, and the total effective rate was up to 93.33% in one study compared with 73.33% for Femoston capsules [45]. Because various herbal mixtures are used, only the most commonly used mixtures are discussed in this section.

### 3.2. Herbal Extract Therapies

Several herbal extracts are commonly used to treat POI in China, including resveratrol, hyperin, and icariin. All of these may affect ovarian cell proliferation, apoptosis, expression of related signal transduction pathways, and local ovarian regulatory factors through multiple pathways, thus playing important roles in the treatment of POI.

#### 3.2.1. Resveratrol

Resveratrol is a natural nonflavonoid polyphenol compound mainly derived from peanut, grape, knotweed, cassia, veratrum, mulberry, and other plants [46]. Studies have confirmed that resveratrol can prevent and treat dementia, osteoporosis, cardiovascular disease, and radiation damage and that it has anti-inflammatory, antitumor, antioxidant, antiaging, and neuroprotective effects [47]. Zeng and Li [48] found that resveratrol can significantly improve triptolide-induced ovarian damage, and Kong et al. [49] found that resveratrol is an effective regulator of ovarian development and oocyte apoptosis. These results indicate that in rat ovaries resveratrol increases the number of resting follicles and total oocytes and reduces the number of developing oocytes and primary follicles. In recent years, studies have shown that SIRT1 can prolong the lifespan of ovaries by inhibiting the apoptosis of oocytes, improving the reserve of primordial follicles, and participating in the regulation of the cell aging process [50], and resveratrol downregulates the expression of Bax, upregulates the activity of SIRT1 and Ku70, and promotes the expression of SIRT1 mRNA to inhibit cell apoptosis [51].

#### 3.2.2. Hyperin

Hyperin is the main flavonoid compound in the kidney-tonifying TCM Cuscutae Semen and is considered to be its main bioactive ingredient [52]. Pharmacological studies have shown that it has antioxidant, antidepressant, and anti-inflammatory effects and has certain therapeutic effects on liver injury, cardio-cerebral ischemia, hypertension, coronary heart disease, and other diseases [53]. Ma and Tan [54] found that after using hyperin gavage treatment for 4 weeks, the body mass and gonadal index of POI mice increased, the pathological damage to the ovary decreased, and the number of follicles at all levels as well as the number of corpora lutea increased. E_2_, AMH, SOD, and catalase levels also increased, and FSH levels decreased. At the molecular level, it is suggested that hyperin improves ovarian reserve in tripterygium glycoside-induced POI mice through the Nrf-2/HO-1 antioxidant stress response and the antiapoptotic effect of the PI3K/Akt pathways.

#### 3.2.3. Icariin

Epimedium, also known as Xianling spleen and which was first published in Shennong's *Herbal Classic of Materia Medica*, is a perennial herb in the Berberidaceae family. Flavonoids are the most important active ingredients of Epimedium and have a variety of biological activities, and icariin is one of the most important [55]. Epimedium is often used to treat female infertility, irregular menstruation, POI, and other diseases, and many studies have shown that icariin can increase alkaline phosphatase activity, reduce tartrate-resistant acid phosphatase activity, and stimulate E_2_ production [55]. The secretion of E_2_ increases, which initiates a feedback loop that inhibits FSH secretion thereby regulating the function of the hypothalamus-pituitary-ovarian axis and promoting the improvement and recovery of ovarian function. In addition, icariin has an estrogen-like effect and can upregulate the expression of estrogen receptors, and it also has a positive effect on the development of rat ovarian follicles [56]. Recent studies have shown that the apoptotic effect of cyclophosphamide on human ovarian granulosa cells depends on the activation of the SMAD/TGF-*β* pathway, while icariin can effectively block the activation of the SMAD/TGF-*β* pathway to reduce the apoptosis of these cells. At the same time, icariin can effectively promote the secretion of E_2_-*β*, a key cytokine, by ovarian granulosa cells [57]. Dong et al. [58] found that icariin can increase SOD levels, increase antioxidant capacity, reduce malondialdehyde production, and reduce lipid peroxide content, suggesting that icariin has antioxidant activity. Its activity in treating POI might be through activation of the PI3K/Akt/mTOR signaling pathway through phosphorylation so as to inhibit oxidative stress-induced ovarian cell apoptosis.

#### 3.2.4. Other Herbal Extracts

Herbal extracts play an irreplaceable role in basic research into POI. For example, quercetin is a flavonoid that promotes follicular development and ovarian granulosa cell proliferation, promotes estrogen secretion, and improves ovarian function in POI mice [59]. Lobetyolin promotes ovarian granulose cells to secrete E_2_, and the possible mechanism is through regulation of the CAMP and P38 MAPK signaling pathways, but lobetyolin does not affect granulosa cell proliferation or differentiation [60]. Moreover, black cohosh extract can relieve the perimenopausal symptoms of POI patients such as hot flashes and night sweats [61]. Herbal extracts are a hot topic in the development of Chinese medicine, but more large-scale clinical studies are needed to clarify its more detailed molecular mechanisms and to apply the research results to the clinic so as to benefit a large proportion of POI patients.

### 3.3. Herbal Patent Medicine Therapies

Herbal patent medicines are herbal products that are processed into various dosage forms based on TCM theory, using Chinese herbal medicines as the raw materials and according to prescribed prescriptions and preparation techniques. Herbal patent medicines have the advantages of stable properties, ease of ingestion, ease of transport, etc., and the most commonly used include Kuntai Capsules (KTC), Yulin Pill (YLP), Bushen Yangjing Granules (BYG), and Yangyin Shugan Capsules (YSC).

#### 3.3.1. KTC

KTC is a commonly used herbal patent medicine for the treatment of POI. It may increase the expression of Smad2/3 and FSH receptors in rat ovarian tissue, thereby increasing ovarian responsiveness to FSH, promoting follicular development, and downregulating the expression of Smad7 in rat ovarian tissue to improve ovarian function [62]. Cui [63] showed that KTC can repair damaged rat ovarian tissue structures, improve ovarian blood supply, promote follicular development, reduce follicular atresia, and increase the number of mature follicles. At the same time, it improved ovarian reserve by increasing the expression of GDF-9 and EGR-1 proteins. Clinical studies have also confirmed that KTC can effectively improve the serum sex hormone levels of patients with POI and can reduce blood lipid levels [18]. A meta-analysis on the effectiveness and safety of KTC in the treatment of POI, including 21 relevant studies covering 1,777 patients with POI, showed that the effective rate and the improvement of serum sex hormone levels were similar to those of western medicine alone. When KTC was combined with western medicine, the effective rate, the Kupperman score, and the improvement in serum sex hormones were better than those of western medicine alone. However, the specific mechanism behind this effect needs further study [64].

#### 3.3.2. YLP

YLP comes from JB Zhang's *Jing Yue's Complete Work* and is often used to treat female infertility and irregular menstruation. In recent years, many studies have shown that YLP has a positive effect on POI patients, and the mechanism might be related to the regulation of mTOR signaling pathway-related genes and their protein expression levels [65]. Yang et al. [66] used a YLP solution to gavage the POI mouse model once a day for six weeks and found that YLP could improve ovarian function by upregulating antioxidant factors and downregulating hypoxia-related factors to improve the ovarian microenvironment and promote follicular development. Another study by their team confirmed that YLP improved oocyte quality by affecting oocytes' mitochondria in POI mice, thus improving ovarian function [67]. An RCT showed that YLP can treat POI more effectively than HRT with a total effective rate of 83.3% vs. 70.0% [19].

#### 3.3.3. BYG

BYG can increase the expression of angiogenesis-related factors (NF-*κ*B, MMP-2, MMP-9, and VEGF), reduce the expression of apoptosis-related factors (caspase-3 and caspase-9), and improve the level of serum E_2_ and AMH, thus promoting the formation of ovarian blood vessels and the development of follicles in the ovarian tissue of a POI rat model [20]. BYG treats POI through multiple functions such as participating in angiogenesis, inhibiting granular cell apoptosis, promoting follicular development, and preventing ovarian interstitial fibrosis [68, 69].

#### 3.3.4. YSC

The pharmacodynamics and pharmacokinetics studies of YSC showed that all compounds displayed strong estrogen-like effects and increased endocrine and antioxidant functions through activation of the aromatase and catalase detoxifying pathways. Furthermore, YSC is involved in prolonging antiosteoporotic effects and delaying the aging of the hypothalamus-pituitary-target gland axis [70]. In a clinical study, Cao and Wang [21] used YSC to treat POI with an effective rate of 93.3%. However, the specific mechanism of YSC remains unclear.

## 4. Acupuncture and Moxibustion Treatment

Acupuncture and moxibustion are methods of preventing and treating diseases and are two of the most ancient practices in TCM. Acupuncture and moxibustion treatments focus on correcting imbalances in Yin Yang and on reinforcing qi and blood to strengthen the body's resistance to and elimination of pathogenic factors. Due to its superiorities, namely, its ease of operation, its low cost, and its satisfactory effect, acupuncture has been used more and more in the treatment of POI. Acupuncture has evolved from the initial forms of acupuncture to now include electroacupuncture, moxibustion, acupoint catgut embedding, acupoint application, auricular point application therapy, transcutaneous electrical acupoint stimulation (TEAS), and acupuncture combined with other therapies [71]. All of these have a positive effect on POI (Table 4). Based on a large number of clinical and animal experiments, the different mechanisms of acupuncture treatment for POI include relaxation of the meridians and promotion of blood circulation, regulation of the hypothalamus-pituitary-ovarian axis, improvement of hormone levels, increasing the production of the antiapoptotic factor Bcl-2, and reducing the production of the proapoptotic factor Bax in ovarian granulosa cells [72–76].

### 4.1. Acupuncture Treatment

Acupuncture refers to using a metal needle to penetrate specific acupoints on the surface of the body and using different techniques to dredge the meridians and collaterals to achieve the purpose of adjusting ying, wei, qi, and blood. Studies have shown the effectiveness of acupuncture and moxibustion in the treatment of POI, and they exert their effects by improving clinical symptoms, slowing down female genital atrophy, regulating hormone levels, and improving pregnancy rates [72, 83]. Wang et al. [84] used acupuncture at different stages in the menstrual cycle to treat POI and showed that it could not only improve the levels of FSH, LH, and E_2_ but could also increase the levels of AMH and increase the AFC in patients with POI. Hui et al. [85] randomized 60 POI patients into a treatment group and a control group. The treatment group was treated with E_2_ and dydrogesterone tablets combined with acupuncture at Guanyuan (RN 4), Zhongji (RN 3), Mingmen (DU 4), Zigong (CA 1), Shenshu (BL 23), Ciliao (BL 32), Guilai (ST 29), Xuehai (SP 10), Sanyinjiao (SP 6), and Taixi (KI 3), while the control group was only given E_2_ and dydrogesterone tablets. After 3 months, they found that acupuncture could increase the efficacy of the medication on improving ovarian function by regulating FSH, AMH, and AFC. Zhuo et al. [77] treated POI with Fang's acupuncture method for regulating menstruation and promoting pregnancy. After three menstrual cycles, the level of FSH was significantly reduced, E_2_ was increased, the endometrium became thicker, and the menstrual symptoms were improved, suggesting that the acupuncture therapy was effective in improving ovarian function and low estrogen symptoms in POI patients.

Acupuncture belongs to the field of TCM and thus follows the principle of syndrome differentiation and treatment. Therefore, the selection of acupoints is extremely important when using acupuncture to treat POI. TCM holds that the main pathogenesis of POI is kidney deficiency and liver depression, and the treatment is mainly based on tonifying the kidney and essence, soothing the liver, and relieving depression [86–88]. We summarized and listed the rules for acupoint selection for POI (Table 5).

### 4.2. Electroacupuncture Treatment

Electroacupuncture therapy is a method to prevent and treat diseases by passing a small amount of current close to the bioelectric currents in the human body through the acupuncture needle, thus adding electric stimulation to the manual stimulation of the needle. Electroacupuncture can adjust human physiological functions, relieve pain, provide sedation, promote blood circulation, reduce muscle tension, and so on [97]. The protective effect of electroacupuncture on the ovary might be by upregulating the expression of the antiapoptotic factor Bcl-2 and downregulating the proapoptotic factor Bax in ovarian granulosa cells [98, 99]. Wang et al. [98] found that electroacupuncture at the Zhongliao (BL 33) and Tianshu (ST 25) acupoints improved ovarian function in POI rats, which may be mediated by regulating insulin-like growth factor 1 receptor (IGF-1R) mRNA expression in ovarian tissue and thus intervening in the IGF-1/IGF-1R axis.

### 4.3. Moxibustion Treatment

Moxibustion involves burning strips or pillars of wormwood over acupoints on the surface of the skin to warm the meridians and reconcile qi and blood. Xiao [78] sought evidence to confirm moxibustion's effect and randomly assigned 66 POI patients to either the moxibustion treatment group or the Femoston control group. The TCM symptom scores were decreased in the treatment group and were less than those in the control group (*P* < 0.05), and the total effective rate of the moxibustion group was 87.1% compared to 80.6% for the Femoston group. Due to the acupoint effect of acupoints, the physical effect of moxibustion, the chemical effect of drugs, and the comprehensive effect of time, it can achieve the effects of tonifying the kidney, warming Yang, regulating menstruation, dispersing cold, and promoting the harmony between Chong and Ren thus attaining a certain clinical effect.

### 4.4. Acupuncture Combined with TCM

Acupuncture combined with TCM in the treatment of POI is a simple and effective treatment method. Compared with the treatments on their own, this combination can improve the clinical efficacy of POI. Li et al. [81, 100] found that acupuncture combined with TCM can effectively improve sex hormone levels and relieve clinical symptoms better than sequential treatment with estrogen and progesterone alone.

### 4.5. Acupuncture Combined with Moxibustion

Acupuncture combined with moxibustion can effectively improve the clinical state of POI patients, adjust sex hormone levels, increase the thickness of the endometrium, and increase the number of sinus follicles to promote the recovery of ovarian function [101–103]. Li [82] found that the clinical symptoms were effectively alleviated and the levels of FSH, LH, and E_2_ were improved through acupuncture combined with ginger-separated moxibustion at the lumbosacral area and acupuncture combined with warm palace moxibustion in the treatment of POI patients.

### 4.6. Acupoint Catgut Embedding

Acupoint catgut embedding is a product of modern technology combined with traditional acupuncture methods. This technique exerts long-lasting stimulatory effects on acupoints to prevent diseases by incorporating absorbable surgical sutures into the acupoints, and it has the advantages of easy manipulation, long-lasting effects, and few toxic side effects. It is widely applicable to various diseases [104], and in recent years, the effectiveness of acupoint catgut embedding in the treatment of POI has become increasingly prominent [105, 106]. Li [107] randomly divided 74 POI patients into an acupoint catgut embedding group and an HRT group. The acupoint catgut embedding group was treated with catgut embedding at Ganshu (BL18), Pishu (BL20), Shenshu (BL23), Guanyuan (CV4), Zhongji (CV3), Sanyinjiao (SP6), and Zigong (EX-CA1) once every 15 days for 6 consecutive treatments, while the HRT group was treated with continuous oral estrogen and progestin for 3 months. The final results showed that acupoint embedding had a more sustained effect on the improvement of hot flashes, sweating, sexual dysfunction, menstrual cycle irregularities, menstrual color, and sex hormone levels in POI patients and that the long-term effects of improving hot flashes, sweating, and menstrual color and increasing E_2_ levels were superior to those of HRT. Lin's clinical study on the treatment of POI by combining TCM for tonifying the kidney and soothing the liver with acupoint catgut embedding with a treatment period of 3 months had a total effective rate of 95%. After treatment, the endometrial thickness and FSH and E_2_ levels were significantly improved. In addition, the results also showed that this combination therapy had few adverse reactions and had a good safety profile [108]. Yang et al. conducted a network meta-analysis of the published literature and found that the efficacy of acupoint catgut embedding is the best among acupoint stimulation therapies, but more randomized controlled trials are needed to verify its efficacy and to promote its clinical application [106].

### 4.7. Acupoint Sticking Treatment

Acupoint sticking treatment is a noninvasive acupoint therapy in which therapeutic drugs ground into a powder are mixed into a paste or a cake and applied on the skin surface at acupoints to treat diseases. Bao [79] divided the patients into a treatment group receiving acupoint application at Qihai (RN 6), Sanyinjiao (SP 6), and Zusanli (ST 36) and a control group treated with oral vitamin E. Both groups were treated continuously for 6 months and stopped treatment during the menstrual period. The results showed that acupoint application could improve hormone levels, and the total effective rate was higher than that of the control group.

### 4.8. Auricular Acupoint Treatment

Auricular acupoints are the acupoints distributed on the auricle, also known as reaction points and stimulation points. When the human viscera or body is sick, there will be local reactions in certain parts of the auricle, such as tenderness, nodules, and discoloration, and these reaction points (ear points) can be stimulated to prevent and treat disease [109]. Qi [80] randomly divided 66 patients into a treatment group treated with auricular acupoints based on TCM at the points for the uterus, ovary, kidney, liver, pituitary, hypothalamus, endocrine, and gonadotropin and a control group treated with pure TCM decoction. The patient massaged the ear points with the paste and pressed the ear points 3–5 times a day for more than 20 s each time until the auricle turned red and hot. The continuous stimulation for 3 months was a course of treatment. The results showed that the total effective rate of auricular plaster therapy was higher than that of TCM alone, and the improvement in FSH and LH in the treatment group was significantly higher than that in the control group.

### 4.9. TEAS

TEAS is a method that combines transcutaneous electrical nerve stimulation therapy with acupuncture points, and it applies pulsed currents to the acupoints through an electrode sheet placed on the skin surface. Compared with traditional acupuncture, it has the advantages of no trauma, less pain, and easy operation. TEAS can increase the number of antral follicles, improve basic endocrine indicators, and improve ovarian reserve function in POI patients, and it has a similar treatment effect as artificial cycle treatment [110]. The mechanism may be through regulation of the expression of FSH receptors on ovarian granulosa cells to promote oocyte development. More clinical trials are needed to verify its efficacy in the future [111].

## 5. Psychotherapy

Psychological stress is one of the main causative factors of POI [112], while, in turn, POI patients suffer from tidal fever and night sweating, irregular menstruation, and infertility, which affect their mental health to varying degrees. Psychotherapy is an important auxiliary method in the treatment of POI, and it can have a positive impact on the treatment of POI [113, 114]. From the perspective of modern medicine, psychotherapy might play a role by affecting the brain center that regulates the hypothalamus-pituitary-ovary axis [115]. Li [116] studied the correlation and distribution between TCM constitution, syndrome types, and anxiety and depression in 120 patients with POI. The results showed that anxiety and depression were common in POI patients and that spleen deficiency and liver depression syndrome were the most closely related to depression. TCM treatments focusing on soothing the liver can effectively alleviate anxiety and depression in POI patients [117]. Xiao et al.'s clinical study showed a significantly higher pregnancy rate in POI patients when supplemented with emotional behavior therapy compared with the control group (73.49% vs. 27.71%) [118]. Because most of the current trials on psychotherapy for POI have been small-scale studies, more large-scale trials are needed to prove its efficacy on POI.

## 6. Dietary Supplements

### 6.1. Medicated Diet

A Chinese medicated diet is a rational combination of different Chinese drugs taken with food under the guidance of TCM theory and is devised using traditional and modern scientific processing technology. The theory of “medicine and food homology” has been known since ancient times in China, and the medicated diet embodies medicine in food, and it increases not only the efficiency of the medicine but also the nutritive value of food, and it can be used to prevent and cure diseases, build up health, and prolong life [119, 120]. Chinese medicine scholar Professor Han followed the ancient admonition and repeated clinical practice to formulate three kinds of medicated diet prescriptions for patients with POI. The prescriptions are (1) swim bladder, pork, medlar, *Pseudostellaria*, and *Rehmannia glutinosa*, (2) *Curculigo orchioides* Gaertn, *Epimedium brevicornu* Maxim, ginger, and mutton, and (3) medlar, green beans, and *Rana dybowskii*. All three diets can tonify the liver and kidneys, improve ovarian function, and relieve the clinical symptoms of POI [121].

### 6.2. Vitamins

#### 6.2.1. Vitamin D

Vitamin D, a precursor of several lipid-soluble steroidal hormones, is involved in the regulation of the female reproductive system in addition to its well-known role in regulating the calcium-phosphorus balance of the blood and bone tissues. Its receptors are widely distributed in the reproductive tissues, including the ovary [122, 123]. Studies have shown that the serum vitamin D concentration has a correlation with antral follicle number, and vitamin D deficiency plays an important role in POI patients [123, 124]. Vitamin D may inhibit the premature activation of primordial follicles by upregulating PTEN, thereby maintaining the balance of the follicular pool and avoiding the occurrence of POI. In addition, it may also inhibit the TLR4/NF-*κ*B signaling pathway to inhibit immune responses and reduce the risk of immune-induced POI [122]. It has been shown that women with POI have decreased bone mineral density due to estrogen deficiency; thus, adequate supplementation of calcium and vitamin D is important to avoid the decline in bone mineral density [125]. In addition, lifestyle changes such as regular exercise, cessation of smoking, and avoidance of excessive alcohol intake are recommended [126].

#### 6.2.2. Vitamin E

Vitamin E is a lipid-soluble antioxidant with *α*-tocopherol as the major form, and it can effectively reverse the adverse effects of oxidative stress on the reproductive system [127]. Increased accumulation of reactive oxygen species during oogenesis is one of the most well-known causes of ovarian insufficiency and decreased ovarian reserve [127–129], and vitamin E, as a cofactor of glutathione peroxidase, plays an important role in scavenging reactive oxygen species in the ovary [130]. Ma et al. found that vitamin E levels are significantly lower in women with POI than in women with normal menstrual cycles [131], and a recent study showed that supplementation with selenium and vitamin E can increase the level of AMH in women with occult POI and can increase the number of antral follicles and the ovarian volume [132].

#### 6.2.3. Vitamin C

Vitamin C (ascorbic acid) is an essential nutrient with an antioxidant effect and can reduce the risk of chronic diseases, and it must be consumed regularly to prevent deficiency [133]. An experiment conducted by Hou et al. showed that vitamin C promoted the proliferation, migration, self-renewal, and paracrine functions of human amniotic epithelial cells in vitro, thus demonstrating the therapeutic potential of these cells in treating POI [134]. Nowadays, many regulatory authorities have increased the recommended intake of vitamin C in their respective countries [135].

## 7. Exercise Therapy

Exercise is a type of physical activity that uses planned, structured, and repetitive limb activities to improve the function of the body as well as the mental health of women with POI. The application of exercise and psychotherapy in POI patients is of great value and can improve the level of sex hormones and clinical symptoms [136, 137]. Women with POI experience significant deterioration in musculoskeletal health due to loss of the protective effects of estrogen, and this deterioration can be delayed or prevented through exercise and resistance training [138, 139]. Additionally, studies have shown that elevated levels of inflammatory cytokines play a critical role in POI [140, 141], and physical activity can reduce the markers of a proinflammatory state [142]. Thus, regular exercise is beneficial for patients with POI. By searching the literature, we also found that some other training methods may also be beneficial to POI patients. For example, Tai Chi, a unique Chinese exercise, can prevent osteoporosis by strengthening the muscles of the lower extremities and can help maintain a sense of balance [143]. Yoga, as a popular physical and mental training method, can alleviate mild vasomotor symptoms and improve sleep [144].

## 8. Conclusions

POI refers to a decline in ovarian function before the age of 40 and is characterized by menstrual disturbances, infertility, and varying degrees of menopausal symptoms. CAM for POI mainly includes herbal products, acupuncture and moxibustion, psychotherapy, dietary supplements, and exercise therapy. Through these therapies, the levels of sex hormones in POI patients can be improved, menstrual cycles can be resumed, menopausal symptoms can be alleviated, pregnancy rates can be improved, and anxiety and depression can be relieved, thereby improving the quality of life of these patients. In conclusion, the active principle of CAM therapies has a strong scientific foundation, and researchers have shown increased interest in this area of medical treatment. Due to the small sample sizes of most studies, however, it is currently difficult to make clear recommendations for clinical guidance. Larger samples and RCTs are needed in the future to verify the efficacy and safety of CAM for the treatment of POI and to provide new strategies for treating POI.

## Figures and Tables

**Table 1:** General view of all therapeutic approaches.

Therapeutic approaches	Specifications	Efficacy	Precautions	Refs.
Herbal products	Appropriate TCM prescriptions are proposed according to TCM doctors' judgment.	Improves hormone levels and relieves clinical symptoms in patients with POI.	Allergies to drugs or contraindications.	[13–68]

Acupuncture and moxibustion	The appropriate acupoints or moxa-moxibustion therapy are chosen based on the disease status of the patient. A typical course of acupuncture is about 30 min, a course of electroacupuncture is about 5–20 min, and a course of treatment with moxibustion is about 40–50 min.	Improves hormone levels and relieves clinical symptoms in patients with POI.	Be careful of fainting conditions.	[69–109]

Psychotherapy	Physicians train, educate, and treat patients to reduce or eliminate their physical symptoms, either verbally or nonverbally.	Relieves clinical symptoms and improves hormone levels in patients with POI.	Be alert for the development of psychological resistance.	[110–116]

Dietary supplements	Different dietary interventions are used to regulate bodily functions and promote health.	Improves hormone levels and relieves clinical symptoms in patients with POI.	Adherence to use should be monitored, but this is not to be an alternative to the usual diet.	[117–133]

Exercise therapy	Planned, structured, and repetitive limb activities are used to improve physical health as well as mental health.	Relieves clinical symptoms in patients with POI.	Avoid excessive muscle fatigue.	[134–142]

**Table 2:** Herbal mixtures for POI treatment in the literature.

Herbal mixture; number of patients (*n*)	Ingredients	Control treatment; number of patients (*n*)	Total clinical effect rate	Model used	Therapeutic effects and actions	Refs.
Bushen Culuan decoction (BCD); *n* = 45	Tu Si Zi, Yin Yang Huo, Xian Mao, Xu Duan, Gou Qi Zi, Nv Zhen Zi, Ze Lan, Pu Huang, Xiang Fu, Chuan Shan Long	E_2_ valerate, clomiphene, and progesterone; *n* = 45	T: 95.35% vs. 88.37%; O: 59.69% vs. 71.32%; P: 27.91% vs. 23.26%	Human study	FSH↓ LH↓ E_2_↑ AMH↑ AFC↑ reduced TCM syndrome scores	[13]
Bushen Huoxue Decoction (BHD); *n* = 20	Shu Di Huang, Shan Yao, Shan Yu Rou, Tu Si Zi, Gou Qi Zi, Dang Gui, Bai Shao, Chuan Xiong, Dan Shen, Xiang Fu, Chuan Niu Xi, Gan Cao	HRT; *n* = 20	T: 100% vs. 70%	Human study	FSH↓ LH↓ E_2_↑ FSH/LH↓ reduced TCM syndrome scores	[14]
Huyang Yangkun Recipe (HYR); *n* = 55	Huang Qi, Dang Gui, Shan Yao, Shu Di Huang, Xian Ling Pi, Tu Si Zi, Sha Shen	Dehydroepi androsterone; *n* = 55	T: 94.55% vs. 85.45%	Human study	Promoted menstruation recovery. FSH↓ E_2_↑ AMH↑	[15]
Erxian Decoction (EXD); *n* = 40	Ba Ji Tian, Xian Mao, Dang Gui, Lu Jiao Shuang, Mu Dan Pi, Zhi Mu, Niu Xi, Huang Bai, Gan Cao, Yin Yang Huo, Chuan Xiong, Shu Di Huang, Nv Zhen Zi, Yi Mu Cao	HRT; *n* = 40	T:95.00% vs. 77.50%	Human study	FSH↓ LH↓ E_2_↑ reduced TCM syndrome scores increased quality of life scores	[16]
Huluan Decoction (HLD); *n* = 30	Du Zhong, Tu Si Zi, Fu Pen Zi, Gou Qi Zi, Dang Shen, Huang Qi, Huang Jing, Bai Zhu, Ju Ye, San Qi Hua, Shi Hu, Yu Zhu, Bai He, Shan Yao, Lian Zi, Hei Dou, Ge Gen, Zi He Che, E Jiao, Gan Cao	Femoston; *n* = 30	T: 83.3% vs. 66.7%	Human study	FSH↓ LH↓ E_2_↓ FSH/LH↓ AFC↑ ovarian volume↑ reduced TCM syndrome scores	[17]

*Note.* T (total effect rate) = number of effective cases/total number of cases, where effective case refers to the patients or animal models whose signs and symptoms were improved after treatment; O: ovulation rate; P: pregnancy rate; E_2_: estradiol; FSH: follicle-stimulating hormone; LH: luteinizing hormone; AFC: antral follicle counts; AMH: anti-Müllerian hormone; TCM: traditional Chinese medicine; HRT: hormone replacement therapy.

**Table 3:** Chinese traditional patent medicines for treating POI.

Chinese traditional patent medicine; number of patients (*n*)	Ingredients	Control sample; number of patients (*n*)	Total clinical effect rate	Model used	Therapeutic effects and actions	Refs.
Kuntai Capsules (KTC); *N* = 50	Shu Di Huang, Huang Lian, Bai Shao, Huang Qin, E Jiao, Fu Ling	E_2_ valerate; *n* = 50		Human study	FSH↓ LH↓ E_2_↑ TC↓ TG↓ LDL-C↓ HDL-C↑	[18]
Yulin pill (YLP); *n* = 30	Shu Di Huang, Tu Si Zi, Dang Gui, Chuan Xiong, Bai Shao, Ren Shen, Bai Zhu, Fu Ling, Lu Jiao Shuang, Du Zhong, Gan Cao, Chuan Jiao	HRT; *n* = 30	T: 83.3% vs. 70.0%	Human study	FSH↓ LH↓ E_2_↑ EM↑ AFC↑ Reduced TCM syndrome scores	[19]
Bushen Yangjing Granules (BYG)	Shu Di Huang, Dang Gui, Bai Shao, Chuan Xiong, Tu Si Zi, Gou Qi Zi, Che Qian Zi, Wu Wei Zi, Fu Pen Zi, Chuan Niu Xi, Xiang Fu, Zhi Qiao, Dang Shen, Yin Yang Huo, Zhi Mu, Yi Mu Cao	Progynova		Sprague–Dawley rat model	NF-*κ*B↑ MMP-2↑ MMP-9↑ VEGF↑ Caspase-3↓ Caspase-9↓	[20]
Yangyin Shugan Capsules (YSC); *n* = 30	Chai Hu, Yu Jin, Bai Shao, Shan Yao, Di Huang, Fu Ling, Xiang Fu	Placebo; *n* = 30	T: 93.3% vs. 63.3%	Human study	FSH↓ LH↓ E_2_↑ reduced TCM syndrome scores	[21]

*Note.* T (total effect rate) = number of effective cases/total number of cases, where effective case refers to the patients or animal models whose signs and symptoms were improved after treatment; E_2_: estradiol; FSH: follicle-stimulating hormone; LH: luteinizing hormone; AFC: antral follicle count; TCM: traditional Chinese medicine; HRT: hormone replacement therapy; VEGF: vascular endothelial growth factor; TC: total cholesterol; TG: triglycerides; LDL-C: low-density lipoprotein cholesterol; HDL-C: low-density lipoprotein cholesterol; MMP: matrix metalloproteinase; EM: endometrium.

**Table 4:** Acupuncture for POI.

Treatment; sample number (*n*)	Control; sample number (*n*)	Total clinical effect rate	Model used	Therapeutic effects and actions	Refs.
Acupuncture; *n* = 30	E_2_ and dydrogesterone tablets; *n* = 30	T: 93.3% vs. 60.0%	Human study	TCM symptom score↓ FSH↓ LH↓ E_2_↑ AMH↑ AFC↑	[77]
Moxibustion; *n* = 31	Complex packing E_2_; *n* = 31	T: 87.1% vs. 80.65%	Human study	FSH↓ LH↓ E_2_↑	[78]
Acupoint sticking treatment; *n* = 35	Vitamin E; *n* = 35	T: 94.29% vs. 71.43%	Human study	FSH↓ E_2_↑	[79]
Auricular acupoint treatment; *n* = 33	TCM decoction; *n* = 33	T: 93.75% vs. 81.25%	Human study	TCM symptom score↓ FSH↓ LH↓ E_2_↑	[80]
Acupuncture combined with TCM; *n* = 86	Artificial menstrual cycle; *n* = 82	T: 86.05% vs. 71.95%	Human study	TCM symptom score↓	[81]
Acupuncture combined with ginger moxibustion; *n* = 20	Acupuncture combined with uterus-warming moxibustion; *n* = 20	T: 75% vs. 77.78%	Human study	TCM symptom score↓ FSH↓ LH↓ E_2_↑	[82]

*Note.* T (total effect rate) = number of effective cases/total number of cases; effective case refers to the patients or animal models whose signs and symptoms were improved after treatment; E_2_: estradiol; FSH: follicle-stimulating hormone; LH: luteinizing hormone; AFC: antral follicle count; AMH: anti-Müllerian hormone; TCM: traditional Chinese medicine.

**Table 5:** The rules of acupoint selection of acupuncture for POI.

Meridian	Acupoints	Refs.
Ren Channel (RN)	Guanyuan (RN 4), Zhongwan (RN 12)	[89, 90]
Du Channel (DU)	Baihui (DU 20), Shenting (DU 24)	[91, 92]
Stomach Meridian of Foot Yangming (ST)	Zusanli (ST 36), Tianshu (ST 25), Guilai (ST 29)	[84, 93]
Shaoyin Kidney Meridian of Foot (KI)	Taixi (KI 3)	[94, 95]
The Spleen Meridian of Foot Taiyin (SP)	Sanyinjiao (SP 6)	[75, 94]
Taiyang Bladder Meridian of Foot (BL)	Shenshu (BL 23), Ciliao (BL 32)	[91, 96]
